# Clinical features and molecular genetic analysis in a Turkish family with oral white sponge nevus

**DOI:** 10.4317/medoral.21437

**Published:** 2018-02-25

**Authors:** Esma Kürklü, Şükrü Öztürk, Andrew J. Cassidy, Gülsüm Ak, Meltem Koray, Kıvanç Çefle, Şükrü Palandüz, Mine G Güllüoğlu, Hakkı Tanyeri, William-Henry-Irwin McLean

**Affiliations:** 1 Department of Oral Surgery, Faculty of Dentistry, Istanbul University, Istanbul, Turkey; 2 Department of Internal Medicine, Division of Medical Genetics, Istanbul Medical Faculty, Istanbul University, Istanbul, Turkey; 3 Tayside Centre for Genomic Analysis, School of Medicine University of Dundee, DD1 9SY, UK; 4 Department of Pathology, Istanbul Medical Faculty, Istanbul University, Istanbul, Turkey; 5 Centre for Dermatology and Genetic Medicine, Division of Molecular Medicine, Colleges of Life Sciences and Medicine, Dentistry&Nursing, University of Dundee, Medical Sciences Institute, Dundee DD1 5EH, UK

## Abstract

**Background:**

Oral white sponge nevus (WSN) is a rare autosomal dominant benign condition, characterized by asymptomatic spongy white plaques. Mutations in Keratin 4 (KRT4) and 13 (KRT13) have been shown to cause WSN. Familial cases are uncommon due to irregular penetrance. Thus, the aim of the study was: a) to demonstrate the clinical and histopathological features of a three-generation Turkish family with oral WSN b) to determine whether KRT4 or KRT13 gene mutation was the molecular basis of WSN.

**Material and Methods:**

Out of twenty members of the family ten were available for assessment. Venous blood samples from six affected and five unaffected members and 48 healthy controls were obtained for genetic mutational analysis. Polymerase chain reaction was used to amplify all exons within KRT4 and KRT13 genes. These products were sequenced and the data was examined for mutations and polymorphisms.

**Results:**

Varying presentation and severity of clinical features were observed. Analysis of the KRT13 gene revealed the sequence variant Y118D as the disease-causing mutation. One patient revealed several previously unreported polymorphisms including a novel mutation in exon 1 of the KRT13 gene and a heterozygous deletion in exon 1 of KRT4. This deletion in the KRT4 gene was found to be a common polymorphism reflecting a high allele frequency of 31.25% in the Turkish population.

**Conclusions:**

Oral WSN may manifest variable clinical features. The novel mutation found in the KRT13 gene is believed to add evidence for a mutational hotspot in the mucosal keratins. Molecular genetic analysis is required to establish correct diagnosis and appropriate genetic consultation.

** Key words:**White sponge nevus, leukokeratosis, oral mucosa, keratins, mutation.

## Introduction

White Sponge Nevus (WSN; OMIM #193900) is a rare, benign autosomal disorder that predominantly affects the oral mucosa and less frequently the mucosal membrane of the esophagus, nasal cavity and the anogenital regions. The characteristic lesions of WSN appear as white or gray coloured, diffuse plaques thickened with multiple furrows. Spongy texture of non-tender and irregular plaques mostly involve the oral mucosa with a predominant distribution of bilateral buccal mucosa, ventral tongue, labial mucosa, alveolar ridges and floor of the mouth (1,2).

The autosomal dominant trait of WSN shows irregular penetrance and variable expressivity, thus familial cases are rare (3). Mutations in the KRT4 or KRT13 genes, encoding mucosa-specific keratin intermediate filament proteins KRT4 and KRT13, respectively, have been shown to be the underlying cause of WSN. All these mutations are heterozygous dominant-negative mutations affecting conserved sequences in the keratin rod domain that are critically important for the assembly of keratin filaments. Failure of this structural scaffold within KRT4- and KRT13-expressing keratinocytes leads to epithelial fragility and hyperkeratosis in the oral and anogenital mucosa. This article describes the clinical and histopathological features of a three-generation Turkish family with oral WSN in relation to the differential diagnosis of oral leukokeratoses. Additionally molecular genetic analysis was performed with the purpose to identify a causative mutation in keratins.

## Material and Methods

- Case History

**Figure 1:**
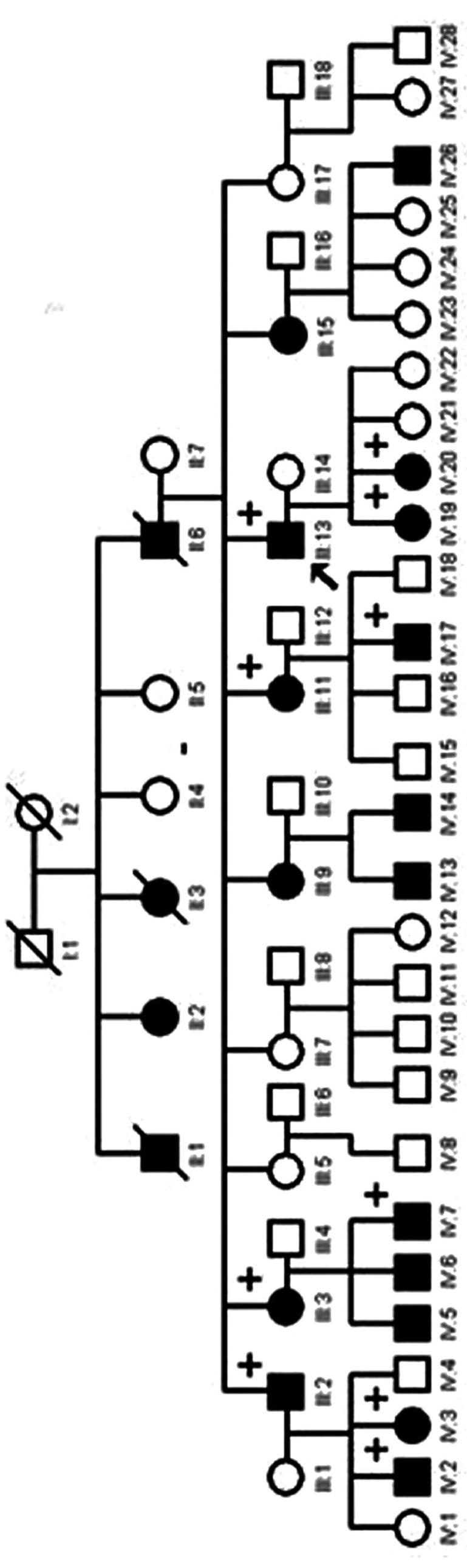
Pedigree of the family. Affected members are indicated by blackened symbols; circles denote females and squares denote males; an arrow appoints the proband; + symbol shows patients that were examined.

The proband of the family is a 36-year-old Turkish male patient (Case I, III:13) who sought consultation for diffuse white coating of oral mucosa which was initially diagnosed as WSN. Detailed family history revealed 19 other affected members of the family 16 alive; (Fig. 1). Other than the proband, nine affected family members were available for clinical examination.

- Light Microscopy 

An incisional biopsy specimen of Case I (III:13) obtained from the right buccal mucosa was embedded in paraffin and stained with hematoxylin and eosin.

- DNA extraction and Direct Sequencing

This is a case-control study and included patients with oral white sponge nevus and healthy controls. The study was performed in the Oral Surgery Department of Faculty of Dentistry, Istanbul University in accordance with Helsinki Declaration of 1975 and as revised in 2013. The patients were informed about the study and all gave consent to participate. For keratin mutation analysis venous blood samples were collected from six affected and five unaffected members of the family and the control group consisting of 48 healthy and unrelated Turkish individuals. DNA was extracted from peripheral blood lymphocytes using the NucleonTM DNA Extraction Kit (Amersham Pharmacia Biotech, Bucks, UK). Primers designed to amplify all coding sequences of the KRT4 and KRT13 genes were used to amplify exonic fragments from genomic DNA. These amplified products were sequenced and the data was examined for mutations and polymorhphisms (GenBank accession number AY043326). Polymerase chain reaction (PCR) was performed in standard buffer containing 1.25 mM MgCl2, 250 µM dNTPs, 20 pmol of each primer, 100 ng of each genomic DNA and 0.5 U of Taq polymerase (Promega, Southampton, UK). The following PCR conditions were used: (94˚C 2 min) x 1; (94˚C 1 min, 60˚C 1 min s, 72˚C 1 min) x 35; (72˚C 5 min) x1. PCR products were purified using the QIAquick® PCR purification system (Qiagen, Crawley, UK) and directly sequenced with the amplification primers and the BigDye® Terminator Chemistry on an ABI3100 Genetic Analyzer (Applied Biosystems, Foster City, CA).

- KRT4 deletion population screening

Exon 1 was amplified using primers KRT4e1.L1 and KRT4e1.R to generate fragments ranging from 447bp to 489bp. PCR was performed in standard buffer containing 1.25 mM MgCl2, 250 µM dNTPs, 20 pmol of each primer, 100 ng of each genomic DNA and 0.5 U of Taq polymerase (Promega, Southampton, UK). The following PCR conditions were used: (94˚C 2 min) x 1; (94˚C 1 min, 60˚C 1 min s, 72˚C 30 sec) x 35; (72˚C 5 min) x1. Products were separated on a 3% agarose gel (Nusieve®, USA) in 0.5x TBE buffer.

## Results

- Clinical findings 

**Figure 2:**
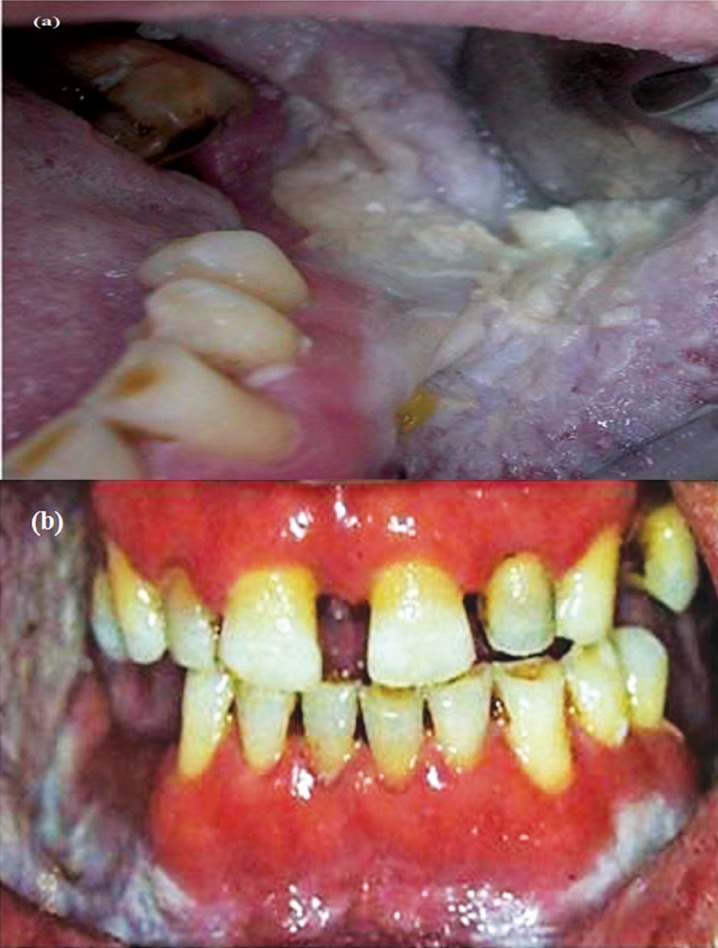
(a & b): Intraoral view of Case 1 with prominent leukokeratotic plaques of the alveolar sulcii and buccal mucosa. Note the apparent sparing of gingiva.

The oral lesions of the proband and the affected members of the family consisted of diffuse, firmly adherent, whitish plaques. Accumulation of the white stringy extensions which elongated to the alveolar crest was more evident in the posterior vestibular sulcii. The keratinized gingiva was spared (Figure 2a & b). Lesions were asymptomatic except mild tenderness to spicy and sour food. In all affected individuals involvement of ventral tongue and a deep median fissure were common findings, with apparently altered texture of the tongue. The patient demographics and clinical findings are shown in  Table 1.

**Table 1:**
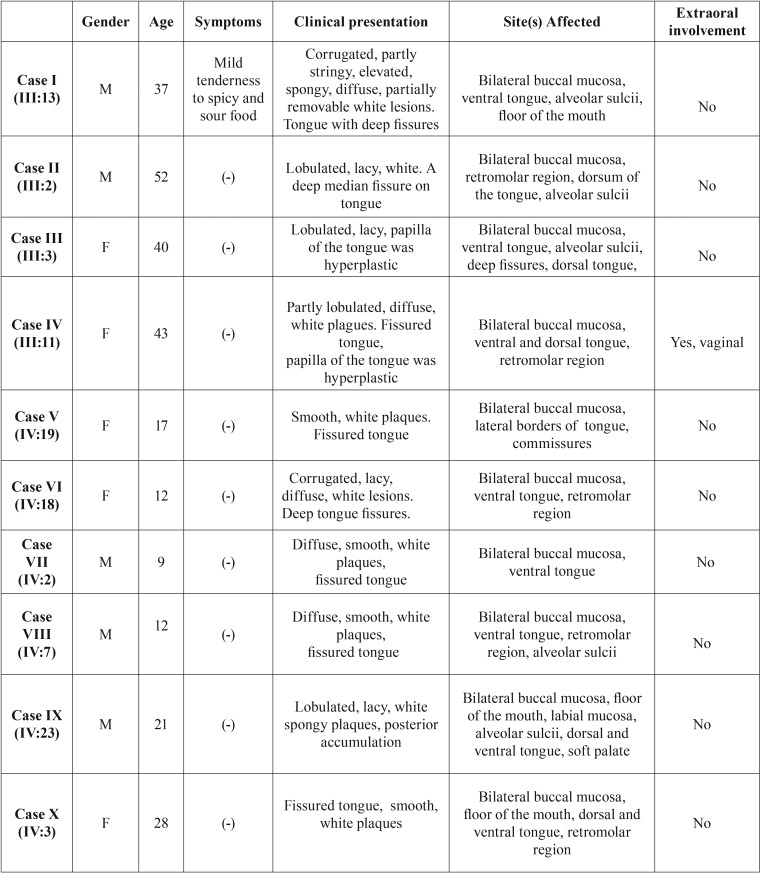
Demographic features and data for clinical assessment.

- Histological Features

**Figure 3:**
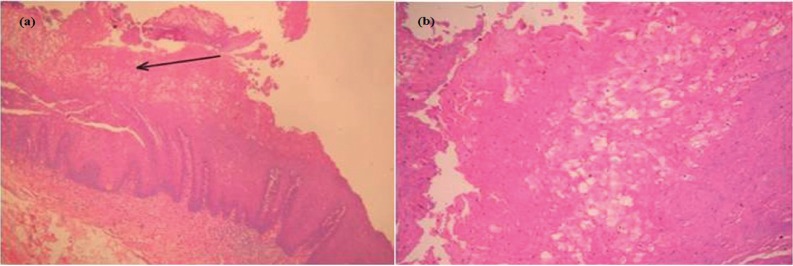
(a&b). The edematous prickle cell layer with cells having condensed pyknotic nuclei (Hematoxylin & Eosin, original magnification x200,400).

The epithelium was acanthotic and hyperchromatic. The cells of the prickle cell layer displayed marked intracellular edema and nuclear pyknosis. Lower half of the epithelium appeared normal. There was no evidence of dysplasia and no basal cell degeneration (Fig. 3a & b).

A novel KRT13 mutation in Case VII (IV:2)

**Figure 4:**
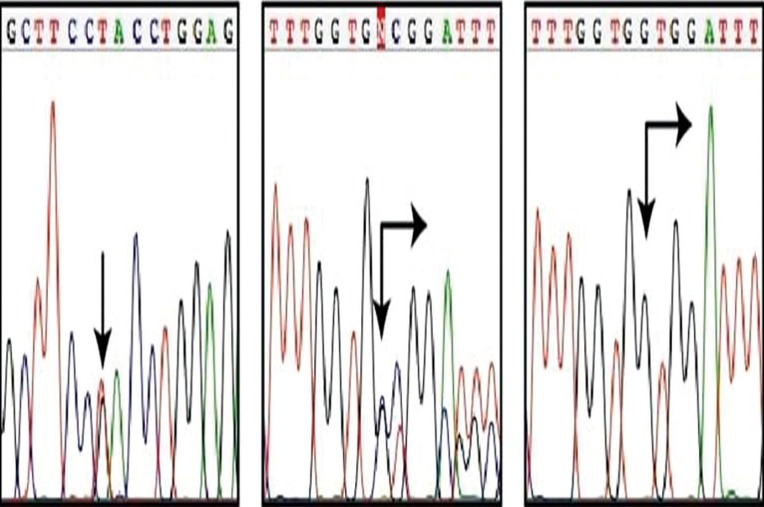
Molecular genetic analysis of K4 and K13 mutations and polymorphisms a. Case VII, carries a heterozygous missense mutation in the KRT13 gene, 352T>G (arrow), predicting amino acid substitution Y118D in the K13 protein (reverse strand sequence data shown). b. Case VII also carries a heterozygous deletion polymorphism in exon 1 of the KRT4 gene, 260-301del42 (arrows), leading to a 14 amino acid deletion in the V1 domain of the K4 polypeptide, 84del14. c. Cloning of PCR products from a heterozygous carrier of the 260-301del42 polymorphism, confirms that the position and extent of this 42 bp deletion (arrows).

Direct sequencing of PCR products generated from the Case VII (IV:2) revealed several previously unreported polymorphisms. In addition, a novel mutation was identified in exon 1 of the KRT13 gene. This mutation is predicted to change codon 118 of the KRT13 coding sequence from tyrosine to aspartic acid. Following the Human Genome Variation Society guidelines for nomenclature, this mutation has been designated 352T>G and Y118D at the DNA and polypeptide level, respectively (Fig. 4a). The mutation was confirmed in all the available affected family members. Further, none of the unaffected family members and 48 control subjects had this mutation.

- KRT4 deletion polymorphism in the control group

Case VII (IV:2) also carried a heterozygous deletion in exon 1 of KRT4. This 42 bp deletion, which was designated as 260-301del42 (Fig. 4b & c), removes a significant portion of the non-helical region between the ISIS motif and H1 domain of the K4 protein, 84del14. This deletion was also found to be a common polymorphism present at a high allele frequency of 30.2% in the control group (in 15 out of 48 individuals) .

## Discussion

The present study demonstrates the clinical and histological manifestations and genetic mutational analysis of a multigenerational familial case of oral WSN. With an onset in childhood, variable expressivity in variable locations, presence of affected individuals in the three consecutive generations (complete penetrance) and mutations in KRT4 and KRT13 the study generates a comprehensive understanding of WSN.

Keratins which consist of intermediate filament proteins type I and type II, are the main structural component of the cytoskeleton in all epithelial cells (4). The basal (proliferative) cell compartment of all stratified epithelia, including the epidermis and mucosa, expresses the basal-specific keratin pair KRT5 and KRT14. Non-keratinizing stratified epithelia, including buccal and anogenital mucosa express KRT4 and KRT13 suprabasally, whereas stratified, cornified epithelia such as the, epidermis, tongue or the palate express KRT1 and KRT10 (5). Mutations in these keratins occur all in the helix boundary motifs, which are recognized mutation “hotspots” in keratin disorders (4,6). These protein sequences are critical for mediating end-to-end associations of keratin subunits during the process of filament assembly. When perturbed by dominant-negative mutations, the intermediate filament cytoskeleton fails to assemble or is severely weakened. When these genetic mutations occur in the genes encoding KRT4 or KRT13, this leads to cell fragility in the oral and/or ano-genital mucosa, which presents as white “spongy” plaques reflecting underlying cell fragility, cytolysis and compensatory overgrowth (hyperkeratosis). This is typical for all keratinocyte fragility syndromes due to mutations in keratins. All mutations identified in KRT4 and KRT13 occur in helix boundary motifs of these proteins. In other keratin disorders mutations have been identified outside these regions in association with milder disease phenotypes (7). To date, therefore only the consequences of severely dominant-negative mutations in WSN were observed and it remains to be seen what visible phenotype, if any, results from mutations outside these ‘hotspot’ regions. In the present study we have idenfied a pathogenic mutation in the KRT13 gene and a number of novel benign sequence variations in KRT4 and KRT13. The previously unreported mutation Y118D in the exon 1 of KRT13 gene is disease causing mutation for this family and adds evidence for a mutation hotspot in the mucosal keratins. This mutation is in the helix boundary motif of the KRT13 protein.

Most of the polymophisms detected in this study occured in introns and are highly unlikely to have any functional consequences. Of the four exogenic variants, three affect amino-acid translation and are located in the VI domain of KRT4 and in the L1 and 1B domains of KRT13. These asymptomatic variants, particularly V187A in helix 1B of KRT13, are suggestive that only highly distruptive mutations located in the helix boundary motifs of KRT4 or KRT13 produce WSN. A particularly interesting polymorphic variant which is a 42 bp insertion-deletion polymorphism designated 260-301del42, in exon 1 was identified in the KRT4 gene of Case VII (IV:2) and ~30% of the Turkish population. This in-frame deletion removes 14 amino acids from the V1 variable domain of the KRT4 polypeptide, 84del14. Similar insertion-deletion polymorphisms have been described in relation to other human keratin genes (8-10). The variable domains are so called since they vary considerably in size and sequence between individual keratins as well as exhibiting inter-species variation (11). Thus, this KRT4 variant is unlikely to have an effect on the normal cellular physiology of mucosal keratinocytes.

The disease is known to occur in a familial fashion with an autosomal dominant trait that shows complete penetrance. The pedigree of this family is also compatible with complete penetrance. All generations had affected members. Although consanguineous marriages are frequent in Turkey, this family had no history of consanguinity. The English literature comprises merely one case of WSN of Turkish origin (12). The present study involves a large familial involvement of three generations with twenty affected individuals.

The diagnosis is based on the clinical appearance of lesions and positive family history. However, in sporadic and mild cases, the diagnosis should be confirmed by histopathological examination. The microscopic features of WSN are characteristic but not necessarily pathognomonic. Epithelial thickening due to hyperparakeratosis, intracellular edema of prickle cells, extensive vacuolisation of the suprabasal keratinocytes and aggregations of keratin intermediate filaments in the upper spinous layers are common features (13). However, eosinophilic condensation in the perinuclear region of cells in the superficial layers of the epithelium, in some instances, is a unique feature to WSN. Similar microscopic findings may be associated with other leukokeratoses like leucodema, pachyonychia congenita, hereditary benign intraepithelial dyskeratosis (HBID) (1). HBID displays conjunctival plaques with hyperaemia concomitant to mucosal white plaques with no involvement of anogenital region. Lichen planus, candidiasis, human papillomavirus infection, verrucous epidermal naevus and hereditary mucoepithelial dysplasia are other white lesions to be ruled out. Eosinophilic condensation in the perinuclear region of cells in the superficial layers of the epithelium, in some instances, is a unique feature to WSN. Morris *et al.* recommended the use of cytologic rather than biopsy specimen with the idea that the latter involves some discomfort to patients and provides essentially no additional information (1,13).

With an onset at infancy or childhood, the disease reaches its peak in early adulthood. In this family the age range displayed a wide spectrum (9-52 years) with disease onset during childhood. A controversy among authors exists about the longevity of the lesions. Some suggest that the disease remains stable throughout life, whereas others suggest persistence with alternate periods of remission and exacerbation (14). The lesions may become more pronounced during pregnancy. The history of Case IV (III:11) was remarkable for a reduction of the white plaques in both oral and anogenital mucosa with the onset of menopause. No other family members had extraoral lesions. This data is consistent with previous reports where extraoral involvement is reported as a rare manifestation.

The disease is largely benign except for the discomfort due to the altered texture of oral mucosa, aesthetic problems and in some cases burning sensation and dry mouth. No standard therapy is known to be completely effective although many attempts including systemic amoxicillin and tetracycline (15,16) and aqueous tetracycline mouth rinse (17) have met varying degrees of success. Therefore, our patients received no treatment. As the lesions persist throughout life and has a benign course, reassurance is all that is required.

This rare disorder may be misdiagnosed, particularly in isolated cases with lesser involvement due to the similarity of clinical and histopathological features to other leukokeratoses of oral mucosa (18). This is of clinical significance in cases where presentation of WSN mimics white potentially malignant lesions, such as leukoplakia, chronic hyperplastic candidiasis, oral lichen planus and dyskeratosis congenita. Liu *et al.* reported four sporadic probands mimicking the clinical and histological manifestations of WSN which were absent from the hereditary and mutational evidences. Hence they suggested performing mutational analysis to reach an accurate diagnosis of WSN (19). In conclusion, the methodology described here will enable a definitive diagnosis of white sponge nevus based on highly reliable molecular genetic data. This has obvious benefits in terms of reassurance to patients and presenting a noninvasive tool when compared with biopsy. Physicians and dentists with comprehensive knowledge of oral mucosal lesions and careful skills to establish an accurate diagnosis would symptomatically -if any exists- treat patients with oral WSN avoiding any unnecessary intervention or therapy and reassure them about the benign character of the lesion.
